# On the Periodical Maturation and Discharge of Ova

**Published:** 1846-07

**Authors:** 


### ECLECTIC DEPARTMENT,

### AND SPIRIT OF THE MEDICAL PERIODICAL PRESS.

---

*On the Periodical Maturation and Discharge of Ova.* By W. M. CARPENTER, A. M., M. D., *Professor in the Medical College of Louisiana.*

The New Orleans Medical and Surgical Journal (March and May, 1846,) contains two articles on the foregoing subject, from the pen of Professor Carpenter, one of the editors of that valuable periodical. The first article is devoted to the subject in its physiological aspect, and presents an excellent compendium of the new facts and views respecting the periodical maturation and discharge of ova, and the connection therewith of the function of menstruation. We had designed to transfer to our columns both articles, but we find they will occupy too much space, and, as we have already, in former numbers of our Journal, presented the more important of the results of late researches, and the physiological deductions to which they have gived rise, we content ourselves with the second article, which treats of the subject in its practical pathological bearings.—*Editor Buff. Journal.*

Viewing menstruation as consisting of two principal acts, the maturation and discharge of ova, and the consequent accession of a hœmorrhage from the uterus, it becomes important for us to determine, in cases of suppression of this function, whether the suppressson is merely of the hœmorrhage, which is the uterine and sympathetic portion of the function, or likewise extends to the ovarian element of the function, the development and discharge of the ova. The determination of this question, even with a degree of probability, would certainly modify our views in regard to the treatment of the cases. If, in any case, it is probable that ova are formed and discharged, and there is suppression merely of the hœmorrhage, with indications that the health of the patient is suffering, in consequence of the suppression, we are justified in the use of aloetics, and other medicines, usually regarded as emmenagogues. But, if the condition of the patient is such, that the ovarian portion of the function—the maturation and discharge of the ovum,—is suppressed, it will be in vain that we resort to these means. We may, in such cases, by irritating the rectum by these means, induce, by proximity, a degree of excitation of the uterine system, and even induce hœmorrhage, but this treatment can avail nothing in correcting the radical lesion, and is frequently most injurious in its consequences.

Unfortunately, we have no means of arriving at a complete and certain diagnosis in these cases; and when the menses are suppressed, we are obliged, in our present state of knowledge, to satisfy ourselves with a degree of probability, as to whether the function is suppressed in all its elements, or merely in its uterine element—its external manifestation.

In forming an opinion on cases of amenorrhœa, we are obliged to rely for our diagnosis principally upon rational signs, and observations of the general condition of the individual, and to keep in view the known effects of such condition, in producing the modifications of the functions under consideration. It is known that, in persons enjoying good health, in plethoric persons generally, and in most acute forms of disease, the ova are periodically discharged, and consequently we may generally infer that the suppression, in such persons, merely extends to a cessation of the elimination of the external discharge, and we adapt the treatment accordingly.

But in many other cases, the general health and condition of the patient interfering with the general nutrition of the ovaries, prevent the fulfilment of the ovarian function, of the production and discharge of ova, and the menstrual function is then suppressed in all of its parts; and the only thing to be done is to repair the condition of the general system, thus restoring the functions of the ovaries, the fundamental and essential part of the menstrual function.

In the autopsy of women who menstruate regulary, and who have died of any, excepting some chronic forms of disease, the ovaries are found to exhibit Graafian follicles, in all states of developement, as well as cicatrices which indicate the continued performance of the ovarian function. But there are some forms of disease which seem especially to affect this function; such are various cachetic conditions in which there is a dimunition of the nutritive qualities of the blood, or which result in great general debility of the system, either of which are incompatible with the performance of the ovarian portion of the menstrual function. In persons who have died in conditions such as these, the ovaries are generally found pale, if not atrophied, with a smooth and even surface, or if any large vesicles are found they are pale, and without the injection which indicates a progressive state; and the cicatrices are either wanting, or of such an appearance as to show that they are not recent. Among the diseases which have been observed to produce this complete suspension of the entire menstrual function are phthisis, chronic bowel complaints, profuse suppurations or other discharges, dropsies, &c., besides various forms of chlorotic disease. Suppression of menstruation being a common concomitant of such affections, and as, in such cases, it has been discovered that the suppression extended to the radical or ovarian portion of the function; and as there is every reason to suppose, by analogy, that those general conditions are competent causes of the suppression, it would always be most safe, in cases of amenorrhœa accompanying such conditions, for us to regard the general condition as the primary one, and the suppression as a consequence. If such be the case, we can only combat the suppression successfully by the employment of such means as will relieve the system of the general condition which has induced the suspension of the menstrual function. Consistently with these views, we find that, in cases of this kind, such remedies act most efficiently as emmenagogues, as have the effect of promoting the general vigor of the economy, and restoring the physiological balance of the functions. General principles will, of course, guide us in this treatment, which must vary according to the indications to be fulfilled. In chlorotic cases, however, the treatment is almost exclusivly *analeptic* or limited to the use of means directed to the promotion of the assimilative functions. Among these means are tonics, but especially the chalybeates a suitable, generally a generous diet, free air and exercise, with the use of any other means which may faciliate the attainment of the same end.

In cases of retarded appearance of the menses, in young persons, and in many cases of suppression, these views will be applicable. Raciborski* observes, that, in these cases, "hygeine affords the best emmenagogues."

It has been frequently observed, that many women were subject to frequent suppressions, and some even did not menstruate at all, but were, notwithstanding, in good health, and retained their fresh and rosy complexion. In these cases the suppression was, of course, limited to the uterine part of the function, and their cessation might or might not require treatment. It is certain, that the common emmenagogues are too indiscriminately used, and are frequently resorted to when nature could have managed the matter as well, or even better. It has been remarked,† that "more than one-half of the Irish and German emigrants who arrive on our shores have amenorrhœa, and it is difficult, through the various treatments adopted, to cause them to return, till they have been residing in this country from six to seven months, when they return naturally."

The same remarks apply equally to many other cases. In such cases I have generally limited my interference to recommending mild purgatives, and hip-baths to be used about the periods when menstruation should come on ; and it is only when other consequences are threatened, that I generally resort to more active means.

The great abuse of emmenagogues has arisen from false views of the nature and relations of the menstrual discharge. Bearing in mind the immense diversity of the causes upon which its suppression may depend, and that the function is a compound one, of which the ovarian part may be performed, while the uterine, or external manifestation, is suppressed, or the suppression may affect the entire function, it is easy to see that the indiscriminate employment of emmenagogues in all cases, must give an unfavorable result.

The function of menstruation has been regarded, and is perhaps still regarded by many, as having for its object the elimination of certain injurious or superfluous materials from the system; and its cessation or diminution would imply, according to those persons, an accumulation of these morbific materiels in the economy. It will be hardly necessary, after the remarks already made, to say any thing in refutation of this opinion. That plethoric

* Raciborski, op. cit.

† Kennedy on Obstetric Auscultation.

persons may be benefitted by, and even require such a drain, might seem possible, were it not for the fact that many women continue plethoric, and nevertheless continue to enjoy good health after the cessation of the menses.

From what I have read, and from my own very limited experience, I would incline to the inference that the large proportion of the serious consequences which are generally attributed to suppressed menstruation might, with much greater justice, be referred to conditions, either local or general, which a nicer discrimination might likewise regard in the light of causes of the suppression. Viewed in this light, suppression would, in many cases, be regarded merely as one of the symptoms of a morbid condition, which, in the progress of its ravages, affected the vital manifestation of the uterine system, in common with those of other organs. The treatment of those primary conditions according to general principles, would be the measure most directly calculated to restore the menstrual function.

Chlorosis has been regarded, by most writers, from the earliest times, as an effect of suppression, or retardation of the appearance of the menses. But when we consider chlorosis in its proper light, that is to say, as a condition characterized by a deficiency in the globules of the blood, the most important of its nutritive elements, and bear in mind the necessary influence of such a condition on the nutrition, and elaborative functions of all the organs of the economy, it is impossible to deny that the suppression or diminution of the ovarian and other reproductive functions is exactly what should be expected from such a condition. But we are not under the necessity of relying exclusively upon theory in this matter, for the pale, smooth condition of the ovaries, and the arrested developement of the Graafian vesicles, in these cases, as described by Raciborski,* leave no doubt that the function of menstruation is affected in its radical and essential part, and that this diminution of function is directly referable to the vitiation of the nutritive qualities of the blood.

The above remarks apply strictly to those cases which depend upon conditions of the system in which a general anœmic or debilitated state affects the menstrual function; but there are many other cases to which the same remarks apply with nearly equal force. There exist various gradations of conditions of the general system, characterized by a tendency to local congestions, or rather by a disturbance of the equilibrium of the circulation, and often partaking more or less of the intermittent type. In conditions of this kind, amenorrhœa generally extends only to the suppression of the uterine discharge, and this suppression is probably due to an undue engorgement of the uterine vessels, which affects the secretory functions of that organ in the same manner as engorgement would affect any other secreting apparatus.

The treatment of such cases, to be decidedly successful, demands that our measures should be directed against the primary condition, rather than directly against the suppression itself. In cases of this kind, the preparations of iron, but especially of quinine, produce the happiest effects. The citrate of quinine and iron is the remedy that seems to be best adapted to such cases. The quinine seems to produce its striking effects in consequence of the powerful influence in relieving local plethora, and

---

* Raciborski, op. cit. 246.

restoring the equilibrium of the general circulation; and when administered about the time the menses should appear, facilitates the performance of the function, by moderating the periodical engorgement of the uterus and restoring it within the physiological or secretory limits. I have used no class of remedies in these, and some chronic engorgements of the uterus, with so much advantage as some of the quinine compounds.

The few remarks that I have made in this paper are by no means intended to cover all cases of amenorrhœa, nor to comment on, or criticise the treatment which has been found, for so long a period, to be so well adapted to many cases, but I wish it to be distinctly understood, that they are made only in reference to the cases and varieties specified.

RELATIONS OF THIS THEORY TO MEDICAL JURISPRUDENCE.

There are cases on record in which the detection of corpora lutea, in post mortem examinations, have been made the basis of criminal prosecution, and have been received in courts of justice as the most direct and satisfactory evidence of seduction or rape, and even as circumstantial evidence of murder committed to conceal these crimes, and avoid their consequences. It is well known, that according to most authorities, up to this time, it has been supposed that an ovum escaped from the ovary, only as a consequence of coition and conception, and the detection of a corpus luteum was regarded, therefore, as a sure indication of conception. Recent researches, by demonstrating the periodical developement and discharge of ova, have shown that the corpora lutea left in cases of fecundation can, by no means, be distinguished from those which remain after the spontaneous discharge of ova. As ova are discharged at each menstrual epoch, we would find them probably after each menstruation.

A recent case occnrred in England, in which a *corpus luteum* was found in the ovary of a woman who had been assassinated, and which gave rise to much discussion as to the value of this fact, and to a controversy upon this subject between Drs. R. Lee and Patterson, which was published in the London Medical Gazette for 1842. the November number, p. 198, and the December number, p. 365.

Cases of this kind are continually liable to occur, from a variety of circumstances. If we were to regard corpora lutea as indicating conception, their detection in post mortem examinations of females might often give rise to or confirm suspicions most unjust to the character of the individual and injurious to others. It would be regarded as evidence of seduction or rape, and the inference that the deceased came to her death by murder or suicide would be a natural, if not an inevitable one. But in accordance with the views already advanced, the existence of corpora lutea indicate, merely, that the woman menstruated at a short period prior to her decease, and is really a sign of no value as regards the probability of conception, or of sexual intercourse.

Another question which sometimes arises in the course of judicial investigation, is, whether a woman may conceive without having menstruated? There are numerous facts of this kind on record, in some of which women bore several children without having ever menstruated; while in one case,*

*See Lancet for 1842, the February number.

the woman, aged twenty-five, bore a child without having ever menstruated previously, but the menses occurred regulary afterwards. These cases are prefectly explained by supposing the ovaries to elaborate and discharge their ova, while the menstrual hæmorrhage alone is suppressed. In order that conception should occur, the ovarian portion of the menstrual function is the only one that is absolutely essential; the menstrual discharge may be absent, without necessarily inducing barrenness.

